# Supplementary material for: Assessment of protocols for characterization of the human skin microbiome using shotgun metagenomics and comparative analysis with 16S metabarcoding
Source: Microbiol Spectr. 2025 Nov 6;13(12):e01732-25. doi: 10.1128/spectrum.01732-25 (PMC12671131; doi:10.1128/spectrum.01732-25)
Supplement: Figures S1 and S2 — Figure S1: DNA quantity distribution in the samples. Figure S2: Number of reads in the samples, without (left) or with MDA (right). [file spectrum.01732-25-s0001.docx]

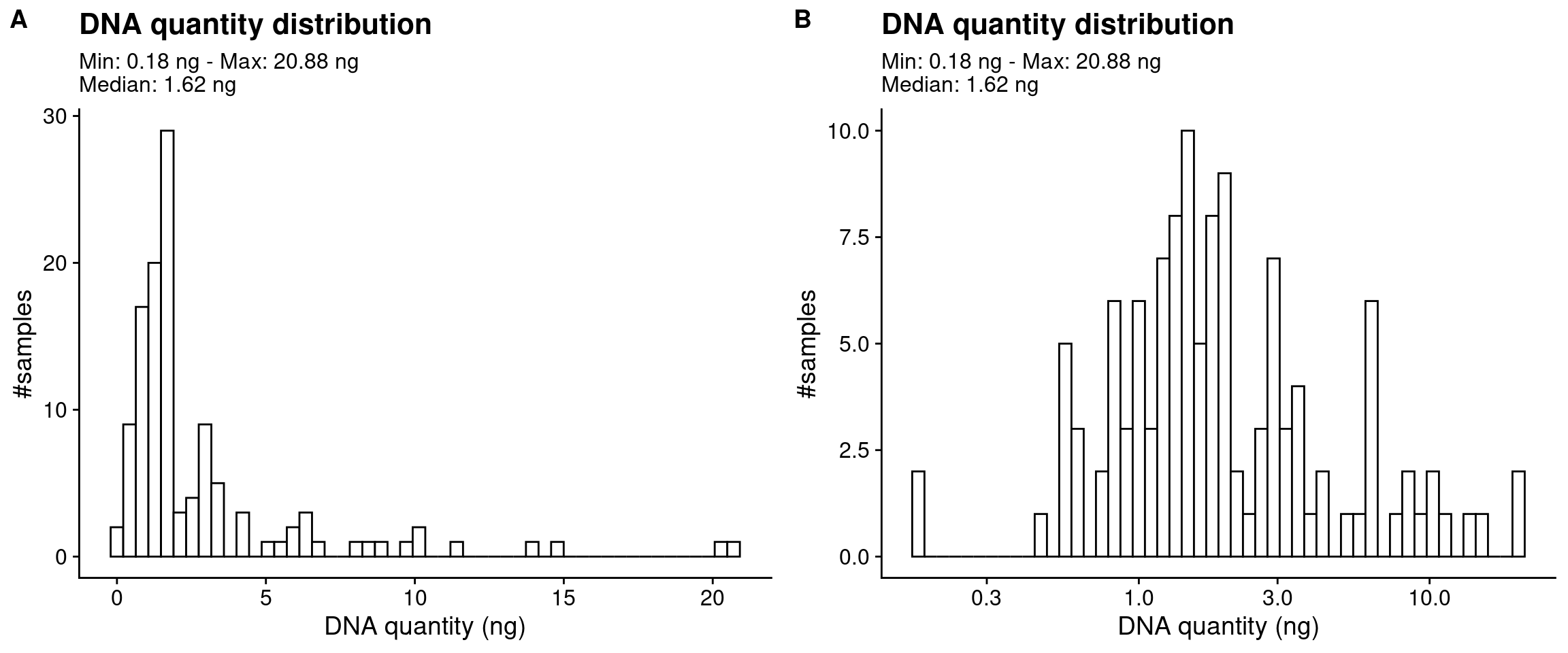


**Supplementary Figure 1.** DNA quantity distribution in the samples.


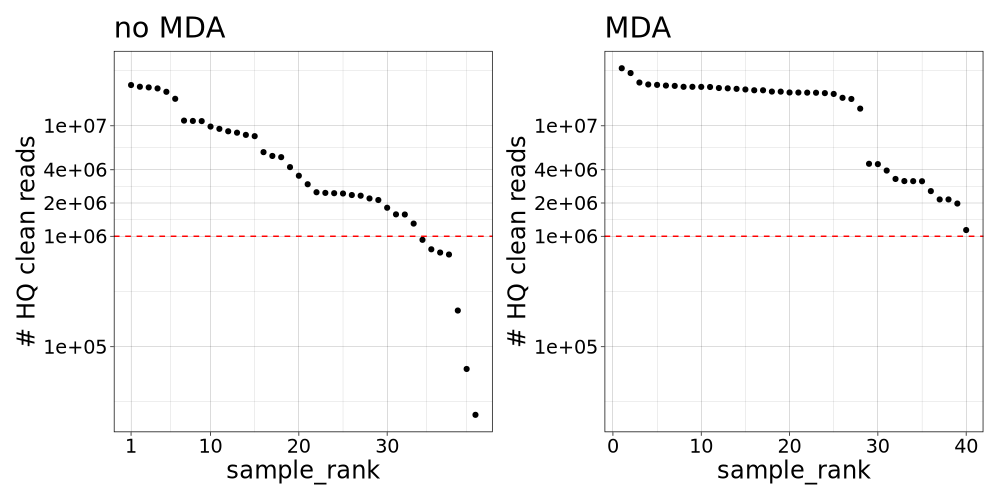


**Supplementary Figure 2.** Number of reads in the samples, without (left) or with MDA (right).
